# Thalidomide for the Treatment of Thrombocytopenia and Hypersplenism in Patients With Cirrhosis or Thalassemia

**DOI:** 10.3389/fphar.2020.01137

**Published:** 2020-07-24

**Authors:** Yaocheng Chen, Ning Cai, Yongrong Lai, Weiwei Xu, Jinyan Li, Lan Huang, Ying Huang, Meiling Hu, Huangju Yang, Jiangming Chen

**Affiliations:** ^1^ Department of Haematology, Wuzhou Gongren Hospital, Wuzhou, China; ^2^ Department of Haematology, The First Affifiliated Hospital of Guangxi Medical University, The First Affiliated Hospital of Guangxi Medical University, Nanning, China

**Keywords:** thalidomide, thrombocytopenia, hypersplenism, thalassemia, liver cirrhosis

## Abstract

Hypersplenism and thrombocytopenia are common complications of liver cirrhosis or thalassemia, but current treatment strategies are limited. This study aimed to evaluate the efficacy and safety of thalidomide in the treatment of hypersplenism and thrombocytopenia in patients with liver cirrhosis or thalassemia. A total of 31 patients with hepatic cirrhosis (n=19) or thalassemia (n=12) diagnosed with hypersplenism and thrombocytopenia (platelet count [PLT] <100×10^9^/L) were included in this prospective cohort study between January 2015 and May 2017. Patients were treated with thalidomide (150–200 mg/d) plus conventional therapy. Spleen length, PLT, leukocyte count (WBC), absolute neutrophil count (ANC), and hemoglobin level (Hb) were measured at baseline, 3, 6, and 12 months. Any adverse events were noted. All of the 31 patients were showed a progressive increase PLT during the 12-month follow-up, and similar results were obtained when subgroup analyses were performed based on the primary disease (cirrhosis or thalassemia). WBC, ANC, and Hb also increased progressively during the 12-month follow-up. Spleen length decreased progressively during the follow-up. No serious adverse events occurred. Thalidomide is a potential treatment for thrombocytopenia caused by hypersplenism in patients with cirrhosis or thalassemia.

## Introduction

Thrombocytopenia (an abnormally low platelet level that results in a hemorrhagic tendency) is a common complication of hypersplenism and is difficult to treat. Hypersplenism and thrombocytopenia frequently develop as a result of other underlying diseases, including liver cirrhosis and thalassemia. Hepatic cirrhosis occurs secondary to a wide variety of disorders such as alcoholic liver disease and infection with the hepatitis C virus (HCV) and/or hepatitis B virus (HBV) (Romanelli and Stasi, 2016). In patients with liver cirrhosis, congestive hypersplenism develops due to portal hypertension, and the resultant increase in blood cell sequestration leads to reductions in the levels of circulating platelets, erythrocytes, and leukocytes (Boyer and Habib, 2015). Additional factors contributing to thrombocytopenia in patients with cirrhosis include decreased thrombopoietin production, bone marrow suppression by viruses (e.g., HCV) or toxic levels of alcohol, and the presence of antiplatelet antibodies (Mitchell et al., 2016; Peck-Radosavljevic, 2017). Thalassemia represents a group of inherited disorders of hemoglobin synthesis that results in defective production of the α-globin or β-globin chains, leading to abnormal erythropoiesis and varying degrees of anemia (Galanello and Origa, 2010). The more severe phenotypes of thalassemia can present with splenomegaly, hypersplenism and thrombocytopenia necessitating transfusion (Taher et al., 2010; Origa and Galanello, 2011; Hashemieh et al., 2012). Thus, despite being disparate disorders, hepatic cirrhosis and thalassemia can both require medical intervention to treat hypersplenism and thrombocytopenia.

Unfortunately, the current treatment options for thrombocytopenia secondary to hypersplenism are limited. Surgical approaches include placement of portosystemic shunts, total or partial splenectomy (via open or laparoscopic procedures), partial splenic artery embolization, and radiofrequency ablation (Zhu et al., 2008; Hashemieh et al., 2012; Boyer and Habib, 2015). Although these surgical techniques have shown efficacy in clinical studies, they are associated with a variety of complications and are not tolerated by some patients. Various pharmacologic approaches have also been utilized, including eltrombopag and romiplostim for patients with cirrhosis (McHutchison et al., 2007; Moussa and Mowafy, 2013) and inducers of hemoglobin F (HbF) synthesis (such as hydroxyurea, butyrate, 5-azacytidine, and decitabine) for patients with thalassemia (Perrine et al., 2005; Olivieri et al., 2011; Musallam et al., 2013; Bayanzay and Khan, 2015). However, medical approaches have shown limited efficacy and are associated with toxicity. Hence, alternative management strategies are needed for thrombocytopenia resulting from hypersplenism.

Thalidomide is an anti-angiogenic, anti-inflammatory, and immunomodulatory drug (Sherbet, 2015) that has been reported to induce HbF synthesis and the proliferation of erythroid cells (Aerbajinai et al., 2007; Moutouh-de Parseval et al., 2008). Two published case reports have described individual patients with thalassemia who responded well to thalidomide (Aguilar-Lopez et al., 2008; Masera et al., 2010). Furthermore, our recent study of nine patients with thalassemia demonstrated that treatment with thalidomide significantly improved the hemoglobin level and reduced spleen size (Chen et al., 2017). Interestingly, we also observed that the platelet levels in two of the nine patients were notably increased after therapy with thalidomide (Chen et al., 2017), suggesting that thalidomide could potentially have utility in the management of thrombocytopenia in patients with hypersplenism.

We hypothesized that thalidomide could be used to treat thrombocytopenia caused by hypersplenism. Therefore, the aim of this prospective cohort study was to evaluate the efficacy and safety of thalidomide in the treatment of hypersplenism and thrombocytopenia in patients with liver cirrhosis or thalassemia.

## Methods

### Study Design and Patients

The ethics committee of Gongren Hospital (Wuzhou City, Guangxi, China) approved the protocol for this study (#2014-003), which was conducted in accordance with the principles of the Declaration of Helsinki. After checking the content of the study, it was found to be in agreement with the Good Clinical Practices. All patients provided informed written consent.

This prospective cohort study included consecutive patients with hepatic cirrhosis or thalassemia (α-thalassemia or β-thalassemia) diagnosed with hypersplenism at Gongren Hospital (Wuzhou City, Guangxi, China) between January 2015 and May 2017. The inclusion criteria were: age >18 years; a diagnosis of liver cirrhosis or thalassemia was made using accepted clinical methods (see below) (Wiegand and Berg, 2013; Brancaleoni et al., 2016); a platelet count <100×10^9^/L before treatment; and either not suitable for splenectomy (for whatever reason) or refused splenectomy after being fully informed of the benefits and risks of surgical intervention. The diagnostic criteria for cirrhosis were: the existence of an underlying disease causing cirrhosis, such as hepatitis B, hepatitis C, or alcoholic liver disease; evidence of portal hypertension, such as the formation of collateral circulation, splenomegaly, or ascites; evidence of liver dysfunction, such as hypoproteinemia, prolonged prothrombin time, metabolic insufficiency, or endocrine dyscrasia; and evidence of hepatic lobe atrophy or liver deformation on computed tomography or B-ultrasound imaging. The diagnostic criteria for thalassemia were as follows: microcytic hypochromic anemia; erythrocyte target cells observed in peripheral blood smears; red blood cells with reduced osmotic fragility; elevated HbF level and slightly elevated HbA2 level (β-thalassemia); and genetic analysis confirmed the presence of a mutant gene associated with α-thalassemia or β-thalassemia.

The exclusion criteria were: previous therapy with erythropoietin, thalidomide, prednisone, androgen, or danazol; severe renal dysfunction; peripheral neuropathy; thrombosis; women who were pregnant or intended to become pregnant in the near future; women who were breastfeeding; and participation in another clinical trial.

### Clinical Assessment Prior to Initiation of Therapy

A venous blood sample was obtained for routine blood tests, including hemoglobin level, platelet count, leukocyte count, and absolute neutrophil count (XN-2000 Hematology Autoanalyzer, Sysmex, Kobe, Japan). Spleen length was measured as the distance from the tip of the spleen to the point at which the midclavicular line intersected with the lower margin of the left rib cage. Spleen length was calculated as the average of the values obtained independently by two physicians using B-scan ultrasonography.

### Interventions

Patients with cirrhosis received standard medical therapy, including antiviral agents (such as interferon or entecavir), hepatoprotective drugs (such as tiopronin, glutathione or diammonium glycyrrhizinate), and medications to improve biliary flow (such as ursodesoxycholic acid). Patients with thalassemia were treated with blood transfusions, visceral organ protection, alkalization, and iron chelation. Patients with a hemoglobin level <60 g/L were given red blood cell transfusion, while those with a platelet count <20×10^9^/L or with hemorrhage received platelet transfusion. Additional therapies included the prevention and treatment of infection and bed rest to reduce the risk of hemorrhage.

All patients received thalidomide in addition to conventional medical treatment. The dose of thalidomide was given with reference to the consensus of Chinese experts on the diagnosis and treatment of primary myelofibrosis (2015 version) (Leukemia, Lymphoma Group CSoHCMA, 2015). Thalidomide was administered before sleep at an initial dose of 50–75 mg/d. If no adverse reactions were observed, the dose was increased the following day to the target dose of 150–200 mg/d, and thalidomide was administered continuously for 1 year.

### Follow-Up and Outcome Measures

All of the patients were followed up for 1 year. The median follow-up time was months (range, to months). The following investigations were carried out at 3, 6, and 12 months after the start of therapy: platelet count, hemoglobin level, leukocyte count, absolute neutrophil count, and measurement of spleen length by B-scan ultrasonography. The requirement for red blood cell transfusion or platelet transfusion during the follow-up period was also analyzed as an outcome measure. For the safety analysis, the occurrence and severity of any adverse events (such as rash, somnolence, constipation, thrombosis, or menstrual disorders) were evaluated. The technicians and assessors evaluating the outcomes were not aware that the patients were being treated with thalidomide.

### Statistical Analysis

SPSS 22.0 (IBM Corp., Armonk, NY, USA) was used for the analysis. Normally distributed continuous variables are expressed as mean ± standard deviation, and non-normally distributed continuous variables are expressed as median (range). For normally distributed data, statistical comparisons were made using analysis of variance and paired t-test. For non-normally distributed data, statistical comparisons were made using Friedman’s analysis of variance and the Wilcoxon signed-rank test. Count variables are expressed as *n* (%). *P* < 0.05 was taken to indicate statistical significance.

## Results

### Baseline Demographic and Clinical Characteristics of the Patients

A total of 31 patients were included in the study (mean age, 44.9 ± 16.0 years; 15 males). The primary disease was liver cirrhosis in 21 patients (67.7%) and thalassemia in 10 patients (32.3%). The baseline demographic and clinical characteristics of the patients are shown in **Table 1**.

**Table 1:** Baseline demographic and clinical characteristics of the study participants.

**Characteristic**	**Value (*n* = 31)**
Age (years), mean ± SD	44.9 ± 16.0
Age (years), median (range)	46 (20–82)
Male, *n* (%)	15 (48.4%)
Primary disease	
Liver cirrhosis, *n* (%)	21 (67.7%)
Thalassemia, *n* (%)	10 (32.3%)
History of red blood cell transfusion, *n* (%)	16 (51.6%)
History of platelet transfusion, *n* (%)	2 (6.5%)
Spleen length (cm), mean ± SD	7.7 ± 3.1
Spleen length (cm), median (range)	7.4 (2.5–13.5)
Leukocyte count **<**4×10^9^/L, *n* (%)	24 (77.4%)
Absolute neutrophil count **<**2×10^9^/L, *n* (%)	19 (61.3%)
Hemoglobin level **<**60 g/dL, *n* (%)	11 (35.5%)
Platelet count (×10^9^/L), mean ± SD	56.4 ± 15.8

SD, standard deviation.

### Requirement for Blood Transfusion During Follow-Up

The proportion of patients requiring red blood cell transfusion during the follow-up period is presented in **Table 2**. A total of 4 patients (12.9%) required red blood cell transfusion during the follow-up period. Very few patients required platelet transfusion during the follow-up period (**Table 2**).

**Table 2:** Numbers of patients requiring transfusion of red blood cells or platelets during the follow-up period.

**Parameter**	**Value (*n* = 31)**
Red blood cell transfusion	
0–3 months	4 (12.9%)
4–6 months	3 (9.7%)
7–12 months	4 (12.9%)
Platelet transfusion	
0–3 months	1 (3.2%)
4–6 months	1 (3.2%)
7–12 months	1 (3.2%)

Data are presented as n (%).

### Platelet Count During Follow-Up

Platelet count during the 1-year follow-up showed an upward trend in the patients treated with thalidomide and was significantly higher at 3, 6, and 12 months than at baseline (all *P* < 0.001; **Table 3**). Similar trends of improvement (i.e., a progressive increase in platelet count during follow-up) were obtained when subgroup analyses were performed based on the primary disease (hepatic cirrhosis or thalassemia; **Table 3**).

**Table 3:** Platelet count during the follow-up period.

	**All patients (*n* = 31)**	**Liver cirrhosis (*n* = 21)**	**Thalassemia (*n* = 10)**
Baseline	56.4 ± 15.8	52.1 ± 12.2	65.4 ± 19.3
3 months	75.1 ± 27.3***	71.3 ± 27.5**	83.2 ± 26.6
6 months	78.9 ± 31.9***	72.7 ± 26.8**	92.1 ± 38.8
12 months	85.8 ± 40.6***	82.3 ± 44.5**	93.1 ± 31.4*

Data are presented as median (range) ×10^9^/L. *P < 0.05, **P < 0.01, ***P < 0.001 vs. baseline value.

### Other Outcome Measures

Leukocyte count was significantly increased at 3, 6, and 12 months as compared with baseline (all *P* < 0.01; **Table 4**). There were also trends toward increases in absolute neutrophil count and hemoglobin level and a decrease in spleen length during the 1-year follow-up, although statistical significance was not attained (**Table 4**).

**Table 4:** Other outcome measures.

**Outcome**	**Value (*n* = 31)**
Spleen length (cm)	
Baseline, median (range)	7.4 (2.5–13.5)
3 months, median (range)	7.3 (1.5–12.7)
6 months, median (range)	6.4 (0.0–11.9)
12 months, median (range)	6.2 (0.0–12.6)
Leukocyte count (×10^9^/L)	
Baseline, median (range)	3.3 (2.0–5.6)
3 months, median (range)	3.3 (1.6–8.2)**
6 months, median (range)	4.2 (1.3–7.5)**
12 months, median (range)	4.4 (1.4–10.5)**
Absolute neutrophil count (×10^9^/L)	
Baseline, median (range)	1.9 (1.0–3.5)
3 months, median (range)	1.8 (0.9–4.1)
6 months, median (range)	2.0 (0.7–4.1)
12 months, median (range)	3.0 (0.8–8.0)
Hemoglobin level (g/dL)	
Baseline, median (range)	69.0 (30.0–147.0)
3 months, median (range)	72.0 (42.0–92.0)
6 months, median (range)	79.0 (42.0–104.0)
12 months, median (range)	80.0 (43.0–96.0)

**P < 0.01 vs. baseline value.

### Safety Analysis

The adverse events reported were rash, somnolence, constipation, and (in females) menstrual disorders (**Table 5**). However, all these adverse events were mild in severity (grade 1 in most cases) and tolerable by the patients. Thrombosis did not occur in any patient.

**Table 5:** Adverse events.

Adverse event	Incidence (*n* = 31)	Severity
Rash	4 (12.9%)	All grade I
Somnolence	6 (19.4%)	All grade I
Thrombosis	0	–
Constipation	11 (35.5%)	8 grade I, 3 grade II
Menstrual disorders^§^	10 (62.5%)	Reduced menstrual volume and delayed cycle

^§^Female patients (n = 16).

## Discussion

The main finding of this study was the cirrhosis or thalassemia patients with thrombocytopenia due to hypersplenism showed a progressive and significant increase in platelet count during 12 months of therapy with thalidomide. Furthermore, leukocyte count also increased significantly during 12 months of therapy with thalidomide, and there were trends toward increases in absolute neutrophil count and hemoglobin level. In addition, there was a numerical decrease in spleen length during the 12-month follow-up. No serious adverse events occurred during the course of the study, and thalidomide was well tolerated. Taken together, our findings suggest that thalidomide may have potential as a novel pharmacologic therapy for thrombocytopenia caused by hypersplenism in patients with cirrhosis or thalassemia. However, prospective, randomized controlled trials will be needed to verify the efficacy and safety of thalidomide in this clinical setting before any recommendations can be made regarding its wider implementation.

The results of our study indicate that thalidomide improved the platelet count in patients with secondary hypersplenism, and this was associated with a progressive but non-significant reduction in spleen size during the 12-month treatment course. We speculate that the changes in spleen length (as well as absolute neutrophil count and hemoglobin level) over time were real but that our study was underpowered to detect these as significant changes due to the small sample size. Taking this into consideration, we suggest that the improvement in platelet count was secondary to a reduction in the degree of hypersplenism, which resulted in decreased sequestration of platelets in the spleen. Indeed, the spleen normally contains around 30% of the body’s platelets, and it is widely recognized that increased platelet sequestration by an enlarged spleen can lead to thrombocytopenia (Smock and Perkins, 2014). The reduction in splenomegaly and hypersplenism would also explain the significant elevation in leukocyte count, apparent improvements in hemoglobin level and absolute neutrophil count (non-significant), and low requirement for red blood cell transfusion that were observed in patients treated with thalidomide. Our findings regarding the possible beneficial effects of thalidomide on hemoglobin level and spleen length are in good agreement with those reported in our previous study of nine patients with β-thalassemia who were treated with thalidomide for 6 months or more (Chen et al., 2017). Two case reports have also described an increase in hemoglobin level in response to thalidomide (Aguilar-Lopez et al., 2008; Masera et al., 2010), consistent with the trends in our observations.

One mechanism by which thalidomide induces the expression of the **γ**-globin gene (and hence HbF) is thought to include increased generation of reactive oxygen species and subsequent activation of p38 MAPK signaling and histone H4 acetylation (Aerbajinai et al., 2007). Furthermore, it has been reported that lenalidomide, a derivative of thalidomide, causes degradation of a transcription factor (IKZF1) (Kronke et al., 2014) that is known to interact with an important transcriptional regulator of the **γ**-globin gene (BCL11A) (Bauer and Orkin, 2015). However, the mechanisms by which thalidomide reduces splenomegaly and hypersplenism remain unknown. There is increasing evidence that angiogenesis and neovascularization play an important role in the development of splenomegaly and hypersplenism. For example, the mammalian target of rapamycin (mTOR) and Janus kinase-2 (JAK2/STAT3) signaling pathways, which are known regulators of angiogenesis, have been implicated in the development of portal hypertension and splenomegaly, and inhibition of these signaling pathways has been shown to reduce splenomegaly (Mejias et al., 2010; Wang et al., 2015; Chen et al., 2016; Wang et al., 2016). Furthermore, it has been reported that elevated levels of vascular endothelial growth factor (VEGF), a critical regulator of angiogenesis and neovascularization, are associated with splenomegaly in patients with chronic myeloid leukemia (Liu et al., 2005) and polycythemia vera (Murphy et al., 2002). Interestingly, previous research has demonstrated that thalidomide can inhibit angiogenesis by suppressing VEGF expression (Komorowski et al., 2006; Maria de Souza et al., 2012; Tan et al., 2012; El-Aarag et al., 2014; Li et al., 2014). Thus, we speculate that the beneficial effects of thalidomide on hypersplenism and thrombocytopenia may be due, at least in part, to a reduction in VEGF secretion and hence suppression of splenic angiogenesis and neovascularization. However, additional research will be needed to establish whether this is the case.

Our safety analysis revealed that thalidomide was well tolerated with most adverse events being grade I in severity, and no severe adverse reactions relevant to thalidomide were observed in this study. It is well known that thalidomide has a teratogenic effect and cannot be used by a pregnant woman (Vargesson, 2015). However, adverse reactions to thalidomide are rare in other patients. Indeed, we observed no adverse reactions to thalidomide in our previous study of patients with thalassemia (Chen et al., 2017). Thus, we believe that thalidomide would be well tolerated by non-pregnant patients with hypersplenism and thrombocytopenia secondary to cirrhosis or thalassemia.

This study has some limitations. First, this was an observational study that may have been susceptible to selection bias, and no control group was included. Therefore, the level of evidence is inferior to that of a randomized controlled trial. Second, this was a single-center study, so the generalizability of the findings is not known. Third, the sample size was small, so the analysis may have been underpowered to detect real differences between time points for some parameters (e.g., spleen length, absolute neutrophil count and hemoglobin level). Nevertheless, a *post hoc* power analysis showed that when using the increase in platelet count at 12 months vs. baseline to calculate the power, assuming that the mean difference in platelet counts was 29.4 ± 37.9 ×10^9^/L, the present study achieved a power of 99% at α=0.05. Fourth, the follow-up period was short, so the longer-term effects and adverse effects of thalidomide were not investigated. Basically, those patients are with conditions (cirrhosis or thalassemia) they will carry for the rest of their lives. Even though the thrombocytopenia and hypersplenism can be managed, they cannot be cured and will recur if the treatments are stopped. In the present study, treatment time was 1 year, but the treating physicians were free to treat the patient as they saw fit afterward. Nevertheless, no follow-up was done beyond the 1-year treatment. The 1-year period was selected for convenience and because previous studies in patients with myelofibrosis showed that the response onset took 1–11 months (Luo et al., 2018) and that the median response duration to thalidomide was 25 or 9 months (Strupp et al., 2004; Luo et al., 2018), but those numbers have to be taken with caution because of the various conditions and combined drugs. The success of thalidomide treatment might lie in fewer transfusions needed and platelet count show effects from treatment, but the relationship with the severity of the underlying diseases was not evaluated. Nevertheless, since this was a preliminary study, the conclusion must be taken with caution, and randomized controlled trials are needed to validate our findings.

Thalidomide is a potential pharmacologic therapy for thrombocytopenia secondary to hypersplenism caused by liver cirrhosis or thalassemia. However, further research is needed to confirm the efficacy and safety of thalidomide in this setting before more widespread use is recommended.

## Data Availability Statement

The raw data supporting the conclusions of this article will be made available by the authors, without undue reservation, to any qualified researcher.

## Ethics Statement

The studies involving human participants were reviewed and approved by Gongren Hospital. The patients/participants provided their written informed consent to participate in this study.

## Author Contributions

JC and WX conceived and supervised the study. JC and YC designed experiments. YC, NC, JL, LH, MH, and HY performed experiments. YH provided new tools and reagents. NC developed new software and performed simulation studies. YH analyzed data. YC and NC wrote the manuscript. YL and JC made manuscript revisions. All authors contributed to the article and approved the submitted version.

## Funding

This study is support by Guangxi Thalassemia Prevention Capacity Improvement Project for funding(2020-1) and Self-funded research project of Health Department of Guangxi Zhuang Autonomous Region (Z2015138).

## Conflict of Interest

The authors declare that the research was conducted in the absence of any commercial or financial relationships that could be construed as a potential conflict of interest.

## References

[B1] Aerbajinai W. Zhu J. Gao Z. Chin K. Rodgers G. P. (2007). Thalidomide induces gamma-globin gene expression through increased reactive oxygen species-mediated p38 MAPK signaling and histone H4 acetylation in adult erythropoiesis. Blood 110 (8), 2864–2871. 10.1182/blood-2007-01-065201 17620452 PMC2018668

[B2] Aguilar-Lopez L. B. Delgado-Lamas J. L. Rubio-Jurado B. Perea F. J. Ibarra B. (2008). Thalidomide therapy in a patient with thalassemia major. Blood Cells Mol. Dis. 41 (1), 136–137. 10.1016/j.bcmd.2008.03.001 18439858

[B3] Bauer D. E. Orkin S. H. (2015). Hemoglobin switching’s surprise: the versatile transcription factor BCL11A is a master repressor of fetal hemoglobin. Curr. Opin. Genet. Dev. 33, 62–70. 10.1016/j.gde.2015.08.001 26375765 PMC4705561

[B4] Bayanzay K. Khan R. (2015). Meta-analysis on effectiveness of hydroxyurea to treat transfusion-dependent beta-thalassemia. Hematology 20 (8), 469–476. 10.1179/1607845414Y.0000000222 25535888

[B5] Boyer T. D. Habib S. (2015). Big spleens and hypersplenism: fix it or forget it? Liver Int. 35 (5), 1492–1498. 10.1111/liv.12702 25312770

[B6] Brancaleoni V. Di Pierro E. Motta I. Cappellini M. D. (2016). Laboratory diagnosis of thalassemia. Int. J. Lab. Hematol. 38 (Suppl 1), 32–40. 10.1111/ijlh.12527 27183541

[B7] Chen Y. Wang W. Wang H. Li Y. Shi M. Li H. (2016). Rapamycin Attenuates Splenomegaly in both Intrahepatic and Prehepatic Portal Hypertensive Rats by Blocking mTOR Signaling Pathway. PLoS One 11 (1), e0141159. 10.1371/journal.pone.0141159 26734934 PMC4703391

[B8] Chen J. Zhu W. Cai N. Bu S. Li J. Huang L. (2017). Thalidomide induces haematologic responses in patients with beta-thalassaemia. Eur. J. Haematol. 99 (5), 437–441. 10.1111/ejh.12955 28850716

[B9] El-Aarag B. Y. Kasai T. Zahran M. A. Zakhary N. I. Shigehiro T. Sekhar S. C. (2014). In vitro anti-proliferative and anti-angiogenic activities of thalidomide dithiocarbamate analogs. Int. Immunopharmacol. 21 (2), 283–292. 10.1016/j.intimp.2014.05.007 24859059

[B10] Galanello R. Origa R. (2010). Beta-thalassemia. Orphanet. J. Rare Dis. 5, 11. 10.1186/1750-1172-5-11 20492708 PMC2893117

[B11] Hashemieh M. Akhlaghpoor S. Azarkeivan A. Azizahari A. Shirkavand A. Sheibani K. (2012). Partial radiofrequency ablation of the spleen in thalassemia. Diagn. Interv. Radiol. 18 (4), 397–402. 10.4261/1305-3825.DIR.4537-11.1 22328281

[B12] Komorowski J. Jerczynska H. Siejka A. Baranska P. Lawnicka H. Pawlowska Z. (2006). Effect of thalidomide affecting VEGF secretion, cell migration, adhesion and capillary tube formation of human endothelial EA.hy 926 cells. Life Sci. 78 (22), 2558–2563. 10.1016/j.lfs.2005.10.016 16310808

[B13] Kronke J. Udeshi N. D. Narla A. Grauman P. Hurst S. N. McConkey M. (2014). Lenalidomide causes selective degradation of IKZF1 and IKZF3 in multiple myeloma cells. Science 343 (6168), 301–305. 10.1126/science.1244851 24292625 PMC4077049

[B14] Leukemia, Lymphoma Group CSoHCMA (2015). [Chinese expert consensus on the diagnosis and treatment of primary myelofibrosis 2015)]. Zhonghua xue ye xue za zhi = Zhonghua xueyexue zazhi 36 (9), 721–725. 10.3760/cma.j.issn.0253-2727.2015.09.001 26462769 PMC7342704

[B15] Li Y. Fu S. Chen H. Feng Q. Gao Y. Xue H. (2014). Inhibition of endothelial Slit2/Robo1 signaling by thalidomide restrains angiogenesis by blocking the PI3K/Akt pathway. Dig. Dis. Sci. 59 (12), 2958–2966. 10.1007/s10620-014-3257-5 25326112

[B16] Liu P. Li J. Han Z. C. Lu H. Wang Y. Xu B. (2005). Elevated plasma levels of vascular endothelial growth factor is associated with marked splenomegaly in chronic myeloid leukemia. Leuk. Lymphoma 46 (12), 1761–1764. 10.1080/10428190500262318 16263579

[B17] Luo X. Xu Z. Li B. Qin T. Zhang P. Zhang H. (2018). Thalidomide plus prednisone with or without danazol therapy in myelofibrosis: a retrospective analysis of incidence and durability of anemia response. Blood Cancer J. 8 (1), 9. 10.1038/s41408-017-0029-4 29335406 PMC5802625

[B18] Maria de Souza C. Fonseca de Carvalho L. da Silva Vieira T. Candida Araujo E. S. A. Teresa Paz Lopes M. Alves Neves Diniz Ferreira M. (2012). Thalidomide attenuates mammary cancer associated-inflammation, angiogenesis and tumor growth in mice. BioMed. Pharmacother. 66 (7), 491–498. 10.1016/j.biopha.2012.04.005 22705333

[B19] Masera N. Tavecchia L. Capra M. Cazzaniga G. Vimercati C. Pozzi L. (2010). Optimal response to thalidomide in a patient with thalassaemia major resistant to conventional therapy. Blood Transfus 8 (1), 63–65. 10.2450/2009.0102-09 20104280 PMC2809513

[B20] McHutchison J. G. Dusheiko G. Shiffman M. L. Rodriguez-Torres M. Sigal S. Bourliere M. (2007). Eltrombopag for thrombocytopenia in patients with cirrhosis associated with hepatitis C. N Engl. J. Med. 357 (22), 2227–2236. 10.1056/NEJMoa073255 18046027

[B21] Mejias M. Garcia-Pras E. Gallego J. Mendez R. Bosch J. Fernandez M. (2010). Relevance of the mTOR signaling pathway in the pathophysiology of splenomegaly in rats with chronic portal hypertension. J. Hepatol. 52 (4), 529–539. 10.1016/j.jhep.2010.01.004 20206401

[B22] Mitchell O. Feldman D. M. Diakow M. Sigal S. H. (2016). The pathophysiology of thrombocytopenia in chronic liver disease. Hepatol. Med. 8, 39–50. 10.2147/HMER.S74612 PMC4847598 27186144

[B23] Moussa M. M. Mowafy N. (2013). Preoperative use of romiplostim in thrombocytopenic patients with chronic hepatitis C and liver cirrhosis. J. Gastroenterol. Hepatol. 28 (2), 335–341. 10.1111/j.1440-1746.2012.07246.x 22849409

[B24] Moutouh-de Parseval L. A. Verhelle D. Glezer E. Jensen-Pergakes K. Ferguson G. D. Corral L. G. (2008). Pomalidomide and lenalidomide regulate erythropoiesis and fetal hemoglobin production in human CD34+ cells. J. Clin. Invest. 118 (1), 248–258. 10.1172/JCI32322 18064299 PMC2117764

[B25] Murphy P. Ahmed N. Hassan H. T. (2002). Increased serum levels of vascular endothelial growth factor correlate with splenomegaly in polycythemia vera. Leuk. Res. 26 (11), 1007–1010. 10.1016/s0145-2126(02)00053-x 12363469

[B26] Musallam K. M. Taher A. T. Cappellini M. D. Sankaran V. G. (2013). Clinical experience with fetal hemoglobin induction therapy in patients with beta-thalassemia. Blood 121 (12), 2199–2212. 10.1182/blood-2012-10-408021 23315167

[B27] Olivieri N. F. Saunthararajah Y. Thayalasuthan V. Kwiatkowski J. Ware R. E. Kuypers F. A. (2011). A pilot study of subcutaneous decitabine in beta-thalassemia intermedia. Blood 118 (10), 2708–2711. 10.1182/blood-2011-03-341909 21700776 PMC3172790

[B28] Origa R. Galanello R. (2011). Pathophysiology of beta thalassaemia. Pediatr. Endocrinol. Rev. 8 (Suppl 2), 263–270. 21705976

[B29] Peck-Radosavljevic M. (2017). Thrombocytopenia in chronic liver disease. Liver Int. 37 (6), 778–793. 10.1111/liv.13317 27860293

[B30] Perrine S. P. Castaneda S. A. Boosalis M. S. White G. L. Jones B. M. Bohacek R. (2005). Induction of fetal globin in beta-thalassemia: Cellular obstacles and molecular progress. Ann. N. Y. Acad. Sci. 1054, 257–265. 10.1196/annals.1345.033 16339673 PMC4261694

[B31] Romanelli R. G. Stasi C. (2016). Recent Advancements in Diagnosis and Therapy of Liver Cirrhosis. Curr. Drug Targets 17 (15), 1804–1817. 10.2174/1389450117666160613101413 27296314

[B32] Sherbet G. V. (2015). Therapeutic Potential of Thalidomide and Its Analogues in the Treatment of Cancer. Anticancer Res. 35 (11), 5767–5772. 10.1016/j.ajog.2003.08.007 26503997

[B33] Smock K. J. Perkins S. L. (2014). Thrombocytopenia: an update. Int. J. Lab. Hematol. 36 (3), 269–278. 10.1111/ijlh.12214 24750673

[B34] Strupp C. Germing U. Scherer A. Kundgen A. Modder U. Gattermann N. (2004). Thalidomide for the treatment of idiopathic myelofibrosis. Eur. J. Haematol. 72 (1), 52–57. 10.1046/j.0902-4441.2003.00188.x 14962263

[B35] Taher A. T. Musallam K. M. Karimi M. El-Beshlawy A. Belhoul K. Daar S. (2010). Overview on practices in thalassemia intermedia management aiming for lowering complication rates across a region of endemicity: the OPTIMAL CARE study. Blood 115 (10), 1886–1892. 10.1182/blood-2009-09-243154 20032507

[B36] Tan H. Chen H. Xu C. Ge Z. Gao Y. Fang J. (2012). Role of vascular endothelial growth factor in angiodysplasia: an interventional study with thalidomide. J. Gastroenterol. Hepatol. 27 (6), 1094–1101. 10.1111/j.1440-1746.2011.06967.x 22098296

[B37] Vargesson N. (2015). Thalidomide-induced teratogenesis: history and mechanisms. Birth Defects Res. C Embryo Today 105 (2), 140–156. 10.1002/bdrc.21096 26043938 PMC4737249

[B38] Wang D. Yin J. Dong R. Zhao J. Wang Q. Wang N. (2015). Inhibition of Janus kinase-2 signalling pathway ameliorates portal hypertensive syndrome in partial portal hypertensive and liver cirrhosis rats. Dig. Liver Dis. 47 (4), 315–323. 10.1016/j.dld.2014.12.017 25637451

[B39] Wang D. Wang Q. Yin J. Dong R. Wang Q. Du X. (2016). Combined administration of propranolol + AG490 offers better effects on portal hypertensive rats with cirrhosis. J. Gastroenterol. Hepatol. 31 (5), 1037–1044. 10.1111/jgh.13207 26487394

[B40] Wiegand J. Berg T. (2013). The etiology, diagnosis and prevention of liver cirrhosis: part 1 of a series on liver cirrhosis. Dtsch Arztebl Int. 110 (6), 85–91. 10.3238/arztebl.2013.0085 23451000 PMC3583179

[B41] Zhu K. Meng X. Li Z. Huang M. Guan S. Jiang Z. (2008). Partial splenic embolization using polyvinyl alcohol particles for hypersplenism in cirrhosis: a prospective randomized study. Eur. J. Radiol. 66 (1), 100–106. 10.1016/j.ejrad.2007.04.010 17532166

